# Can the ability to adapt to exercise be considered a talent—and if so, can we test for it?

**DOI:** 10.1186/s40798-017-0110-3

**Published:** 2017-11-29

**Authors:** Craig Pickering, John Kiely

**Affiliations:** 1 Institute of Coaching and Performance, School of Sport and Wellbeing, University of Central Lancashire, Preston, UK; 2 Exercise and Nutritional Genomics Research Centre, DNAFit Ltd, London, UK

## Abstract

Talent identification (TI) is a popular and hugely important topic within sports performance, with an ever-increasing amount of resources dedicated to unveiling the next sporting star. However, at present, most TI processes appear to select high-performing individuals at the present point in time, as opposed to identifying those individuals with the greatest capacity to improve. This represents a potential inefficiency within the TI process, reducing its effectiveness. In this article, we discuss whether the ability to adapt favorably, and with a large magnitude, to physical training can be considered a talent, testing it against proposed criteria. We also discuss whether, if such an ability can be considered a talent, being able to test for it as part of the TI process would be advantageous. Given that such a capacity is partially heritable, driven by genetic variation between individuals that mediate the adaptive response, we also explore whether the information gained from genetic profiling can be used to identify those with the greatest capacity to improve. Although there are some ethical hurdles which must be considered, the use of genetic information to identify those individuals with the greatest capacity appears to hold promise and may improve both the efficiency and effectiveness of contemporary TI programmes.

## Key points

1. Talent identification programmes often identify those with the greatest current ability, as opposed to the greatest capacity to improve.
2. This capacity to improve is linked to physical adaptation to exercise, which is partially genetically mediated.
3. Genetic profiling holds promise in being able to identify those individuals with the greatest capacity to improve, as well as the best methods through which to yield these improvements.

## Background

The accurate identification of youth sporting talent has, in recent decades, emerged as a hugely important and yet controversial topic [1, 2]. Interest in talent identification (TI) is illustrated by a growing academic literature [1–3], along with a number of best-selling popular-science books on the topic [4–7]. Traditionally, sporting TI programmes have, through a mix of subjective and objective tests, sought to identify young athletes with “talent,” using this identification as a prediction of adult performance. However, despite the massive allocation of resources into the identification and development of young talent, it remains unclear whether or not early TI processes are either empirically justified or practically effective.

One fundamental limiting factor is that physical performance tests employed to discern between those who have the talent to excel in the future, and those who do not, actually only provide a snapshot of current abilities. The subsequent logical leap is the presumption that those who perform well at that given time are most likely to be successful as adults. Yet, due to the inherently non-linear complex nature of biological maturation, these performance snapshots offer inherently poor predictive value. As an illustration, within athletes competing in the 2012 Olympic 100 m final, personal bests at age 18 ranged from 10.27–10.48 s. In comparison, one of this paper’s authors (CP) ran 10.22 s at this age, faster than all the finalists. Yet, whilst these athletes progressed to achieve multiple sub-10s 100 m times, CP peaked at 10.14 s.

The reasons why CP, along with countless other high-performing juniors, did not maintain their relative world standings are obviously complex, varied and multifactorial [8, 9]. This illustrates the gross inaccuracies associated with current approaches to predicting future senior potential based on youthful performance. Similarly, where TI processes have been empirically evaluated, these inefficiencies remain, with fewer than 2% of athletes identified as having the potential to be elite within a school sports programme winning senior international medals [10].

Despite these inefficiencies, however, clubs and organizations invest exorbitant sums on TI and development initiatives in the hope of unearthing future talent. Manchester City’s Academy programme, for example, reportedly costs £12 million per year [11]. Yet such large investment is perceived as both economically feasible and justified by the occasional unearthing of exceptional talent; 15 Manchester City Academy graduates have been capped at senior international level, and one, Shaun-Wright Phillips, was sold by the club for £21 million.

A clear limitation of the TI process is that, during maturation, current performance is not directly indicative of future potential. In fact, no standard physical assessment provides insight into how an individual is likely to respond to future training. In this article, we explore the possibility that the utilization of genetic markers associated with the capacity to favorably respond to imposed training stress may provide valuable, and currently missing, insights relating to future trainability, rather than current ability, thus providing clues as to whether the athlete has the innate “talent” to respond to training.

## The hereditary aspect of talent

A standardized, widely accepted definition of talent is hard to find. A review of the complexities surrounding an adequate definition of talent is beyond the scope of this article; however, Issurin recently utilized a broad definition of talent as “a special ability that allows someone to reach excellence in some activity in a given domain” [2]. In conceptualizing this definition, Issurin leaned heavily on Howe and colleagues [12], who proposed that talent has five properties: it is partially innate; its full effect may not be evident at an early stage; it has early indications that provide a basis for predicting who might excel; only a few possess it; and it is domain specific.

Implicit within any definition of talent is the assumption that it is at least partially genetically determined. This is most obvious when considering the physiological underpinnings of elite performance, all of which are, to some degree, genetically influenced. Approximately 50% of baseline maximal oxygen uptake (VO_2max_) is heritable [13], as is 45–99.5% of muscle fibre type [14, 15]. Muscle strength is estimated to be ~ 52% heritable [16]. Anthropometric qualities, often used as TI indicators, are also genetically mediated, with height approximately 80% heritable [17]. So too are non-physical traits associated with elite performance; for example, stress resilience has a genetic component [18, 19], as does motivation to exercise [20]. All of these findings suggest that talent is at least partially mediated by genetic factors. Indeed, de Moor et al. reported that 66% of the variance in elite status is heritable [21].

Whilst elite athlete status appears to have a strong genetic component, to date, it remains apparent that the available genetic information is insufficient to reliably predict those most likely to reach elite status in the future. Gene variants (polymorphisms) most frequent in elite athletes appear to hold little to no predictive ability on their own. For example, a single nucleotide polymorphism (SNP) in *ACTN3*, a gene encoding for a protein found in fast-twitch muscle fibres, is associated with elite sprint athlete status [22]. Here, between 97 and 100% of elite sprinters have at least one R allele, making the XX genotype rare [23]. However, the fact that at least some elite sprint and speed-power athletes have the XX genotype [24] illustrates that it perhaps lacks the sensitivity required to correctly identify talent. In addition, approximately 82% of the world’s population possess at least one R allele [25], thereby illustrating its lack of discriminatory power in discerning between potential athlete and non-athlete.

The inability of single SNP to effectively discriminate between eventual phenotype has led to the suggestion that utilizing a panel of SNPs, each associated with a physical capacity deemed contributory to elite performance, may provide greater predictive ability. Using such an approach, a total genotype score (TGS) is calculated, with a higher TGS indicative of a greater chance of achieving elite status. This approach has had some success, with mean TGS in athlete groups greater than controls [26, 27], although it does not yet appear to distinguish between competitive levels within athlete groups [28]. Again, however, the sensitivity and specificity are not sufficient to rule out false positives (identifying someone as a future athlete who is later unsuccessful in this endeavor) or false negatives (identifying someone as a future non-athlete, who goes on to become a world-class athlete). As such, the current consensus is that genetic testing has no role to play in the TI process [29, 30], although this opinion is formed on the assumption that elite athletes have common genotypes.

## Discussion

### Is the ability to adapt to exercise a talent?

Whilst traditional TI programmes attempt to identify future elite performers through the application of physical, psychological and subjective evaluations, it is not clear whether this is the best approach. One issue with the use of such performance tests is that they measure the current status of the athlete, as opposed to the potential for that athlete to improve and develop. Consider the use of a 60-m sprint test in order to identify talented sprinters in a cohort of 15-year-olds. Whilst the test is valid and will accurately identify the quickest athletes, it is not clear that the fastest athletes at age 15 will be fastest at age 25. There is, therefore, a mismatch between what the test measures—current ability—and the TI processes goal—identifying future ability [31]. Instead, the focus of the TI process should be to find individuals with the potential to develop their skills and physiology in order to become successful senior athletes [31], commonly referred to as talent development (TD).

**Fig. 1:**
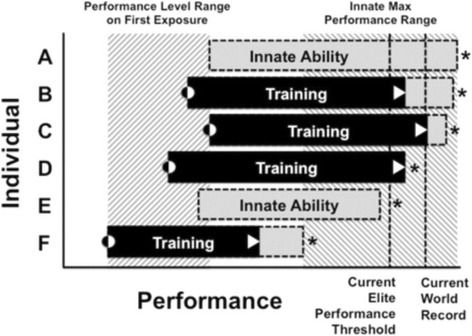
A theoretical model illustrating inter-individual variation in performance and potential (reproduced from [8]). Here, six individuals (A–F) have differing initial performance levels (performance level range on first exposure); F has the lowest, and C has the highest. The individuals also have different ceilings to his/her performance (innate max performance range), with A having the highest potential. However, training represents the journey from baseline potential to final potential; A and E do not train, and so will never reach their ceiling. Whilst C is the current world record holder, B has the potential to outperform them—but only if B can maximize their training to drive the required response. Legend: Max = maximum; asterisk = maximum performance threshold for each individual; triangle = current performance level; black-white circle = initial performance level. Reproduced from [8]. Permission has been granted for reproduction

TI programmes, therefore, should attempt to identify those with the greatest ability to develop, provided that their maximal ability is sufficient to be an elite athlete. This fits into a model proposed by Tucker and Collins [8], detailed in Fig. 1, whereby athletes have different baseline abilities that reflect the untrained state, but also different maximal abilities, which represent the performance ceiling for each athlete. There is not necessarily a relationship between the two; an athlete with a high start point might have a low ceiling. Conversely, an athlete with a low start point might have a higher ceiling. In this model, what becomes key is the potential of the athlete to improve with training—and whether they maximize this potential. For exercise adaptation to be considered a talent, it needs to fit the following five criteria proposed by Howe and colleagues [12].

### Is exercise adaptation partially innate?

An ever-increasing body of research now suggests that genetic factors modify the adaptive response to exercise. The seminal research in this regard is the HERITAGE (Health, RIsk factors, exercise Training and GEnetics) Family Study, in which sedentary adults undertook a 20-week aerobic exercise training programme. The mean post-intervention improvement in VO_2_max in this cohort was 384 mL O_2_ min^−1^. However, some subjects saw no improvement, whilst others exhibited much larger improvements than the mean, as high as 1100 mL O_2_ min^−1^ [32]. Genetic factors accounted for almost 50% of this inter-individual variation [33]. Genetic association studies also show the modifying impact of single SNP on exercise adaptation. For example, R allele carriers of *ACTN3* appear to show greater improvements in power following a strength training intervention than X allele carriers [34, 35]. It is clear that exercise adaptation is partly genetically driven, and is therefore innate.

### Are the full effects of this talent not fully evident at an early age?

Growth, maturation and the physical development of youth athletes are non-linear in nature [36, 37]. Children and adolescents are physically less able than adult elite athletes due to differences in muscle size, strength [38, 39] and energy system development [40, 41], which may limit the magnitude and type of adaptations that are possible [1, 42]. This was illustrated by Radnor et al. [43], who reported that maturation modified the adaptive response to resistance and plyometric training in a group of adolescent males. Based on these findings, it appears that knowledge of the full ability of a person to be able to adapt to exercise is likely not fully understood until maturation has occurred [42], fulfilling this talent criterion.

### Are there early indications of this talent?

This is perhaps the most difficult question to answer as part of these criteria. In part, this is due to a lack of research examining the magnitude of exercise adaptation in youths, and comparing that to either the magnitude of adaptation in those same youths as adults, or associating that adaptive response with sporting success later in life. There are responders and non-responders to specific training interventions in youths [43, 44], but it is not clear how this affects adaptation in adulthood. Nevertheless, the ability to adapt favorably to exercise as a youth will positively impact development by taking the athlete from their baseline towards their performance ceiling, increasing the possibility of adult success.

### Do only a minority of people possess this talent?

Overwhelmingly, research suggests that almost everyone has the ability to adapt to exercise, with the small number whom show no improvements labelled as non-responders [45]. However, emerging research suggests such exercise non-response abates with modification of training parameters, such as an increase in training intensity [46] or frequency [47]. However, the magnitude of training response differs between individuals. As detailed earlier, this was apparent in HERITAGE, with a mean post-training VO_2max_ improvement of 19%, although some subjects exhibited improvements of less than 5%, and others improvements of > 40% [48]. Similar wide-ranging magnitudes of adaptation have been reported after strength training and combined strength and endurance training [49–51]. It appears that, whilst almost everyone exhibits positive adaptations to exercise, those of the greatest magnitude are limited to a smaller number of individuals, a hallmark of a talent.

### Is this talent domain specific?

Whilst genetic variation exhibits a modifying effect on exercise adaptation, the final point to consider is whether this is global (i.e. all types of exercise) or modality specific (i.e. individuals exhibiting large resistance training adaptations do not necessarily exhibit the same adaptive magnitudes to aerobic training). As previously discussed, the *ACTN3* R allele is associated with greater improvements in muscle phenotype following resistance training [34, 35]. However, regarding VO_2max_ adaptation, the X allele appears to be associated with larger improvements [52], illustrating that the genetic predisposition to exhibit a greater adaptive response is domain specific. Karavirta et al. [51] randomized subjects to receive strength training only, endurance training only, concurrent strength and endurance training or no training. Within each group, subjects exhibited the expected range of adaptation; however, in the concurrent training group, no subject was in the highest quintile of improvement for both VO_2peak_ and maximal voluntary contraction, again indicating that an ability to respond aerobically is separate to the ability to respond to strength training. It appears, therefore, that the ability to adapt favorably to exercise is specific to particular domains, as opposed to a global ability.

### Can we consider exercise adaptation a talent?

Exercise adaptation is a highly complex and individualized process, mediated by genetic, environmental and epigenetic factors [53]. The influence of variation at the genetic level accounting for large amounts of the inter-individual adaptive response to exercise is clear [32, 45, 53], allowing the conclusion that the magnitude of adaptation is partially innate. It is also domain specific, with those possessing the ability to exhibit large improvements following one type of training not guaranteed to exhibit improvements of the same magnitude following a different modality [51]. The presence of a small number of individuals who have very large post-training improvements in a physical trait [48] illustrates that only a few possess this ability. The ability to exhibit large adaptations to exercise is also potentially masked by maturation effects. So far, there is a paucity of evidence examining whether those athletes who are highly adaptable during their youth remain so during their adult years. Nevertheless, based on the evidence available, it does appear that the ability to respond favorably, and with a large magnitude, to exercise can be considered a talent.

### Can we test for this talent?

Traditional TI processes appear to identify athletes who are already more able than their peer group, as opposed to those who represent the greatest ability to improve. The ability to test for this latter trait would therefore enhance the TI process, providing some predictive measure as to the future level of the athlete. As Abbott and Collins [31] state, successful prediction of future accomplishments requires identification of characteristics indicating that an individual has the potential to both develop in sport and become a successful senior athlete. Crucially, recent research suggests that individuals respond optimally to different types of training [44, 54, 55], illustrating that being able to match promising youngsters with the training type most likely to elicit the greatest improvements could be invaluable. This can reduce the trial-and-error process, increasing the time period available for an athlete to maximize their potential by minimizing ineffective and inefficient training methods.

Since the ability to respond to exercise is partially mediated by genetic factors, being able to test for these factors holds promise. Given that the impact of any one SNP on this process is likely to be small, a more promising approach is the use of whole genome or large (> 600,000) SNP sequencing. A small number of studies have used this process, with early evidence suggesting they could have some predictive ability [44, 56]. This process is separate from the use of genetic testing to identify the commonly held definition of sporting talent—adult performance—whereby promising athletes’ genetic profiles are compared to a pool of elite athletes to look for commonalities, the assumption being that a greater number of commonalities is associated with a greater chance of being elite. At present, there is no evidence to support this [29]. Indeed, it is likely that different genes impact baseline ability (what is commonly identified in traditional TI processes) and ability to adapt to exercise, as detailed in Fig. 1. Certainly, a greater body of research is required before evidence-based guidelines for the use of genetic testing to support talent development (as opposed to pure TI) can be utilized, but these early findings hold promise. Given the issues discussed within the current TI process, it could be argued that anything that improves the current offering should be utilized.

In addition, there are a host of ethical questions that surround genetic testing, not just within sports, but also public health [57]. For example, is it acceptable to test under 18s, who may not have the required maturity to both fully understand the results in context and give informed consent? What happens if, as part of a routine genetic test, a disease-associated SNP is discovered? To avoid this, should such gene variants be removed from the testing panels? Can clubs insist that their contracted players must undertake a genetic test, and who owns that data when the test is completed? The resolution of such considerations is a challenge to the translation of laboratory-based genetic research to the field, but they are related to how the information is presented and interpreted, as opposed to whether genetic information should or should not be used.

## Conclusion

Whilst widespread across sport, traditional TI processes have a number of inherent problems. Perhaps the biggest issue is that they appear to identify current ability, as opposed to future potential, a fact which is not helped by the poor predictive ability of currently used tests of talent. Instead, TI programmes might be better placed to identify youngsters with the greatest capacity to improve, which is partially comprised of the ability to adapt to exercise. As genetic factors account for approximately 50% of the variation in adaptation to exercise, profiling to uncover these genetic underpinnings could be a useful future adjunct to the TI process, and also allow for athletes to undertake training that they are more likely to see favorable adaptations to, creating a personalized training process making athletes more likely to achieve their potential. With the many inefficiencies and high costs associated with TI, it is clear that we only need to be marginally better at the TI process in order to be disproportionately more effective at developing talent, and genetic testing potentially represents this marginal gain. Within this paper, we have focused on the physiological aspects of talent and talent identification. It is, however, worth noting that sporting prowess is not depending solely on physiology, and a number of psycho-emotional and cognitive traits are also associated with athletic achievement. Such traits include, for example, innate stress resilience, and a host of attitudinal factors, such as motivation, perseverance and personality dispositions [2, 3, 58]. Importantly, as with other phenotypes, these capacities are also partially mediated by hereditary influences and partly by life history [59, 60]. In summary, the ability to positively respond to the training stimuli imposed by physical exercise fulfils the required criteria to be considered a talent. The emergence of genetic testing may enable the more accurate identification of athletes who, thanks to a favorable genetic profile, possess a heightened ability to exhibit the greatest responses to training, thus improving the efficiency and efficacy of the talent identification process.
